# The *Psathyrostachys juncea DWARF27* gene encodes an all-*trans*-/9-*cis*-beta-carotene isomerase in the control of plant branches in *Arabidopsis thaliana* by strigolactones

**DOI:** 10.1093/g3journal/jkae147

**Published:** 2024-07-15

**Authors:** Xiaomin Ren, Qian Ai, Zhen Li, Qiao Zhao, Lan Yun

**Affiliations:** College of Grassland, Resources and Environment, Inner Mongolia Agricultural University, Hohhot 010018, China; College of Grassland, Resources and Environment, Inner Mongolia Agricultural University, Hohhot 010018, China; College of Grassland, Resources and Environment, Inner Mongolia Agricultural University, Hohhot 010018, China; Shenzhen Key Laboratory of Synthetic Genomics, Shenzhen Institute of Advanced Technology of Chinese Academy of Sciences, Shenzhen, Guangdong 518055, China; College of Grassland, Resources and Environment, Inner Mongolia Agricultural University, Hohhot 010018, China; Key Laboratory of Grassland Resources Ministry of Education, Inner Mongolia Agricultural University, Hohhot 010011, China

**Keywords:** *DWARF27*, *Psathyrostachys juncea*, *Arabidopsis thaliana*, functional orthologue, tillers/branches number, strigolactones

## Abstract

Strigolactones (SLs), carotenoid-derived plant hormones, govern the growth and development of both monocotyledonous and dicotyledonous plants. DWARF27 (D27), a plastid-targeted protein located at the initiation site of the core pathway in SL synthesis, plays a crucial role in regulating plant tillering (branching). In rice (*Oryza sativa*) and wheat (*Triticum aestivum*), OsD27 and TaD27-B proteins modulate the number of plant tillers by participating in SL biosynthesis. Similarly, AtD27 in *Arabidopsis thaliana* is required for SL production and has a significant impact on phenotypic changes related to branching. At the same time, *TaD27* in wheat has been confirmed as a functional orthologue of *AtD27* in *Arabidopsis*, and both *Psathyrostachys juncea* and wheat belong to the *Triticeae*, so we speculate that *PjD27* gene may also have the same function as *AtD27* in *Arabidopsis*. In this study, we initially screened the *PjD27* gene significantly associated with tillering regulation through transcriptome data analysis and subsequently validated its expression levels using qRT-PCR analysis. Furthermore, we conducted phylogenetic analysis using amino acid sequences from 41 species, including *P. juncea*, to identify closely related species of *P. juncea*. Here, we analyze the conservation of D27 protein among *P. juncea*, rice, wheat, and *Arabidopsis* and provide preliminary evidence suggesting that PjD27 protein is an orthologue of D27 protein in *Arabidopsis*. Through reverse genetics, we demonstrate the crucial role of *PjD27* in regulating plant branching, establishing it as a functional orthologue of *D27* in *Arabidopsis*. Furthermore, following transient expression in tobacco (*Nicotiana tabacum*), we demonstrate that the subcellular location of the PjD27 protein is consistent with the cellular location of TaD27-B in wheat. Quantitative analysis of SLs shows that *PjD27* is a key gene regulating tillering (branching) by participating in SL biosynthesis. By elucidating the function of the *PjD27* gene, our findings provide valuable genetic resources for new germplasm creation and improving grain yield in *P. juncea*.

## Introduction

Tillering is one of the major components affecting plant yield. Generally, agronomists consider tiller number and tiller angle as the most important tillering traits in crop plants (Ishikawa *et al.* 2005; Dong *et al.* 2023; Wang, Tu *et al.* 2023). Fertilization controls plant growth parameters such as tiller number, leaf size, and panicle number. However, several genes are known to influence tillering. For example, the key gene regulating the number of tillers/branches in wheat (*TN1*) is highly conserved across wheat varieties. The *TN1* gene was cloned from the wheat *tn1* mutants and is known to impact the yield of wheat by promoting the growth of wheat tiller buds (Dong *et al.* 2023). Similarly, the transcriptional regulation of fertilizer-responsive gene *Os1900* in rice (*Oryza sativa* L.) can mediate the increase of tiller number and grain yield (Cui *et al.* 2023). Tiller angle is also important and can improve photosynthetic efficiency and increase field density by affecting the plant architecture to increase grain yield per unit area (Zhao *et al.* 2023). Genes play a role in tiller angle, and the *Tiller angle control 1* (*TAC1*) gene is a key gene known to regulate the dynamic expression of tiller angle during plant growth stages in rice (Wang, Tu *et al.* 2023).

Strigolactones (SLs) are plant hormones that regulate plant growth and development (Proust *et al.* 2011). SLs inhibit shoot branching in plants of many different species. SLs inhibit auxin synthesis in plant lateral branches and then indirectly act on apical dominance (Agusti *et al.* 2011). For example, *Arabidopsis thaliana* treated with SLs produced fewer branches, an effect attributed to inhibition of auxin synthesis (Brewer *et al.* 2009).

SLs have likely evolved to mitigate undue plant growth when nutrients are limited. Early work reviewing rice, observed that the level of SL production is inversely related to phosphorus availability, inhibiting the growth of rice tiller buds when nutrients are limited (Umehara *et al.* 2010). *RMS3* and *RMS4* in pea (*Pisum sativum*) shoots inhibit the outgrowth of pea buds by participating in the biosynthesis and feedback signal of SLs (Dun *et al.* 2009). Similarly, *D17*, *D3*, and *D27* are key genes in the biosynthesis or signaling pathway of SLs in rice, and their defective mutants have an increased number of tillers due to the lack of this SL-mediated axial synthesis inhibition (Cui *et al.* 2023).

DWARF27 (D27) is a plastid-targeted protein required for the biosynthesis of SLs, which converts all-*trans*-β-carotene into 9-*cis*-β-carotene during the biosynthetic pathway of SLs, and further forms the precursor of SLs known as carlactone through the catalysis of Carotenoid Cleavage Dioxygenase 7 (CCD7) and CCD8. Finally, SLs are formed through other reaction paths, thereby inhibiting the number of plant tillers/branches (Brewer *et al.* 2009; Alder *et al.* 2012; Waters *et al.* 2012; Harrison *et al.* 2015). Some studies have revealed the regulatory function of *D27* in plant tillering (branching). Rice *d27* mutants were associated with high tillering and severe dwarfism (Lin *et al.* 2009; Wang and Li 2011). In the case of calcium deficiency, the level of SLs in rice will decrease accordingly, thereby stimulating the outgrowth of tiller buds (Shindo *et al.* 2018). The *Arabidopsis d27*-1 mutant had a phenotype of significantly increasing the number of axillary buds. When the exogenous SL analog GR24 was applied, the shoot branching was significantly inhibited in *Arabidopsis* mutant (Waters *et al.* 2012). The *TaD27* genes which are orthologues of rice *D27* in SLs biosynthesis were identified in wheat. *TaD27-*RNAi plants had an increased number of branches or tillers, and *TaD27-B-*overexpressing (*TaD27-B-*OE) plants had fewer tillers (Zhao *et al.* 2020).

Natural grassland in high-latitude cold steppe regions faced degradation tendency due to overgrazing and climate change (Li, Peng *et al.* 2022). Divers of *P. juncea* varieties with good tolerance and high biomass are urgently needed (Fikadu *et al.* 2022; Main *et al.* 2023). *Psathyrostachys juncea* (Fisch.) Nevski, as a high-quality cool season forage grass with good tolerance, has a high breeding value in forage yield improvement (Liu *et al.* 2006). The study of its tillering-related genes contributes to the production of high-yield and grazing-tolerant *P. juncea* varieties. In addition, the increase in *P. juncea* biomass yield contributes to soil surface coverage and ecological restoration (Li *et al.* 2023), and provides potential genetic resources for improving the tillering traits of other perennial grasses (Li, Yun *et al.* 2022).

Wheat is one of the most significant cereal crops, which is utilized as a staple food for human consumption and contributes about 20% of proteins worldwide (Shiferaw *et al.* 2013; Yeni and Teoman 2020). *P. juncea* with abundant superior germplasm of stress-bearing and disease resistance, is a wheat-related wild, which provides an excellent gene pool of wheat (Wang *et al.* 2016; Hu *et al.* 2018). However, the yield traits of *P. juncea* still need to be improved (Li, Yun *et al.* 2022). Therefore, we used *P. juncea*, a perennial bunch grass, as the research material to analyze the function of tillering-related genes, aims to improve the yield of *P. juncea*.

We screened candidate genes involved in the SL synthesis pathway that are highly correlated with tillering through transcriptome data analysis, and the candidate gene *PjD27* was selected as the main focus of our research (Li, Yun *et al.* 2022). The subcellular location of PjD27 protein was analyzed by transient transfection of tobacco (*Nicotiana tabacum*), and the *35S:pCambia1300*-*PjD27-cYFP* vector (Supplementary Fig. 1, a and b) was constructed to infect *Arabidopsis* by *Agrobacterium* infection. We observed that infection of *A. thaliana* (Col-0) with the associated gene led to a significant reduction in the number of branches, though the *Arabidopsis d27* mutant (*At1g03055*; SALK_071443.2) restored the number of branches associated with the wild type, consistent with the effect of *AtD27* on the phenotype of *Arabidopsis* (Waters *et al.* 2012). Our results indicate that *PjD27* is the main gene regulating tillering behavior in *P. juncea*, providing valuable information for alleviating the lack of germplasm resources and improving the yield of *P. juncea*.

## Materials and methods

### Plant materials and treatment conditions

Columbia wild-type *A. thaliana* (Col-0) and tobacco materials were kindly provided by the Shenzhen Institute of Advanced Technology (SIAT), Chinese Academy of Sciences. The *Arabidopsis d27* mutant (At1g03055, SALK_071443.2) was acquired from AraShare Science. Primers LBb1.3 (5′-ATTTTGCCGATTTCGGAAC-3′), d27-Left Primer (5′-ATCTTCGATCTCTGGAGGCTC-3′), and d27-Right Primer (5′-GGATACGGCAACTAGGGTTTC-3′) were designed by T-DNA Primer Design (http://signal.salk.edu/tdnaprimers.2.html). Both *Arabidopsis* and tobacco were cultivated in a 1:1 mixture of peat soil and vermiculite. *Arabidopsis* was grown under controlled conditions with a constant temperature of 23°C, a light cycle of 16 h, followed by an 8-h dark period. Tobacco plants were cultured at 25°C with a light cycle of 12 h, alternating with a dark period of equal duration.

### RNA sequencing

Thirty dense tillering (DT) plants and another thirty sparse tillering (ST) plants were selected as materials in *P. juncea*, with 3 biological replicates for DT and ST plants, respectively. These plants were planted in the forage germplasm nursery of Inner Mongolia Agricultural University in Hohhot, Inner Mongolia, China. Plants were grown in the forage nursery for a period of 2 months before they were destructively sampled. The tiller nodes were meticulously cleaned and subsequently frozen using liquid nitrogen. Total RNA was extracted using a total RNA extraction kit (DP432, TransGen, Beijing, China), and cDNA was synthesized from the total RNA using PrimeScript RT reagent Kit (AW311-02, All-Gold, Beijing, China). The resulting cDNA library was then sequenced by Biomarker Technologies (Beijing, China). The sequencing data were screened to analyze gene expression levels, which were calculated using Fragments per Kilobase of transcript per Million mapped reads (FPKM). In addition, the false discovery rate was used as a key indicator for differentially expressed genes (DEGs) screening to calculate the significance of differences in gene expression levels. Subsequently, the gene expression heatmap was generated using the online platform EHBIO (http://www.ehbio.com/ImageGP/index.php/Home/Index/PHeatmap.html). Pathway analysis of DEGs was conducted based on the KEGG (Kyoto Encyclopedia of Genes and Genomes) database (Supplementary Fig. 3). For detailed test conditions, review our previous publication (Li, Yun *et al.* 2022).

### Sequence and phylogenetic analysis of D27

The coding sequence (CDS) of the *PjD27* gene was successfully cloned and subsequently subjected to sequencing analysis by Ruibiotech Company, located in Guangzhou. Homologous sequence alignment was performed using the BLAST tool (https://blast.ncbi.nlm.nih.gov/Blast.cgi) in the NCBI database, and the amino acid sequences of D27 proteins derived from 41 species were used for phylogenetic analysis. A neighbor-joining (NJ) phylogenetic tree was constructed using Interactive Tree of Life (iTOL) online platform (https://itol.embl.de/) and Molecular Evolutionary Genetics Analysis 11.0 software (Zhu *et al.* 2022) with 1,000 bootstrap test replicates. Conserved domains within D27 proteins from *P. juncea*, wheat, rice, and *Arabidopsis* were analyzed using the Structure tool at NCBI (https://www.ncbi.nlm.nih.gov/Structure/cdd/wrpsb.cgi), while the amino acid sequences were compared utilizing DNAMAN software.

### Generation of *35S:PjD27 Arabidopsis* plants

Total RNA was extracted from the tiller nodes of *P. juncea* individuals using an RNA extraction kit (DP432, TransGen, Beijing, China). Subsequently, cDNA was generated from 1 µg of total RNA by PrimeScript RT reagent Kit (AW311-02, All-Gold, Beijing, China). The full-length CDS of the *PjD27* gene was first amplified using *D27*-F (5′-ATGGCCTTGATGCCCGTGG-3′) and *D27*-R (5′-GTTCTGAATTTTGGGACATATTGCAGC-3′) primers designed in SnapGene 6.0.2, with 2 × Phanta Max Master Mix enzyme (Vazyme, Nanjing, China) and cDNA as a template. Then, it was ligated to the Blunt vector using a cloning kit (CB501-01, TransGen, Beijing, China), and positive clones were confirmed by sequencing. Approximately 0.5 µL of the cloning products was then reamplified using pCambia-1300-cYFP Recombinant primers *1300*-*D27*-F 5′-CCGAATTCGGAGTCGACACTAGTATGGCCTTGATGCCCGTGG-3′ and *1300-D27*-R 5′-TCCACCTCCGACCGGTGCACTAGTGTTCTGAATTTTGGGACATATTGCAGC-3′ (the underlined parts are the adapter primers of the pCambia-1300-cYFP vector). The full-length CDS was then transferred to pCambia-1300-cYFP (an infection vector provided by SIAT) via recombination, generating the final *35S:PjD27* vector. The Col-0 and *Arabidopsis d27* mutant were then transformed by the floral dip method. Transgenic seedlings were grown in 16 h light for 11 days on one-half-strength Murashige and Skoog medium containing hygromycin B (50 mg.ml^−1^) and termentin (100 mg.ml^−1^).

### Western blotting analysis

The leaves of the *Arabidopsis d27* mutant, Col-0, and positive plants were pulverized into a fine powder using liquid nitrogen and subsequently transferred to a centrifuge tube containing an equal volume of 2 × SDS loading buffer. After spotting, electrophoresis was performed at a voltage of 120 V for a duration of 1 h. Subsequently, the gel was inverted and positioned beneath the PVDF membrane. It was then covered with filter paper and a sponge before being transferred to the membrane using an electric field strength of 100 V for a period of 60 min at a temperature of 4°C. After incubation of the PVDF membrane with the primary antibody GFP and subsequent treatment with the secondary antibody [HRP-labeled goat anti-mouse lgG (H + L)], dropwise addition of a mixture containing an equal amount of BeyoECL Moon A solution and B solution was performed, followed by imaging using a western blot (WB) exposure instrument (Ren *et al.* 2023). The aforementioned reagents were supplied by SIAT.

### RT-PCR and RT-qPCR analysis

The roots, tillering nodes, and leaves from 32-day-old *Arabidopsis d27* mutants, Col-0, transgenic lines expressing *PjD27* under the control of the CaMV 35S promoter with both Col-0 background [*35S:PjD27* (Col-0)] and *Atd27* mutant background [*35S:PjD27* (*d27*)], were used as experimental samples for subsequent analysis using RT-qPCR and RT-PCR techniques. Moreover, the freeze–dried tillering nodes from DT and ST were also included as other samples for subsequent RT-qPCR experiments.

In all cases, total RNA was extracted from the aforementioned materials using a total RNA extraction kit (DP432, TransGen, Beijing, China). cDNA was synthesized from the total RNA using PrimeScript RT reagent Kit (AW311-02, All-Gold, Beijing, China). Various template cDNAs were diluted into 4 concentration gradients (1, 1/2, 1/5, 1/10), and RT-qPCR was performed using Taq Pro Universal SYBR qPCR Master Mix enzyme (Vazyme, Nanjing, China) with a volume of 5 µL. Cycle conditions were as follows: 95°C for 2 min; then 40 cycles of 95°C for 5 s, 60°C for 30 s; 95°C for 15 s; followed by melt-curve analysis. Primer pairs for quantitative RT-PCR were designed using NCBI (https://blast.ncbi.nlm.nih.gov), except for *ACTIN* (*At5g09810*) (Waters *et al.* 2012) and *ACTIN-Pj* (Li, Yun *et al.* 2022). All quantitative RT-PCR primers are listed in Supplementary Table 2. *PjD27* gene expression in *Arabidopsis* was quantified based on the 2^−ΔΔCt^ method. RT-PCR primer pairs used for identifying *35S:PjD27* (Col-0) and *35S:PjD27* (*d27*)-positive plants were designed using NCBI. The primers were RT-*D27*-F 5′-AGAAGTTCGCCGGAAGGAAGGAAG-3′ and RT-*D27*-R 5′-TGGGACATATTGCAGCGGAG-3′, resulting in a total product size of 579 bp. The *ACTIN* (*AT3G18780*) was provided by SIAT.

### Subcellular location analysis of PjD27 protein

The recombinant fusion protein *35S:PjD27*, along with the negative control *35S:cYFP*, was propagated through *Agrobacterium* GV3101 and subsequently injected into the dorsal surface of 1-month-old tobacco leaves. YFP (Yellow Fluorescent Protein) fluorescence was captured using a Nikon (A1R) confocal microscope after 2 days of tobacco growth (Bian *et al.* 2023). The subcellular location of the PjD27 protein was analyzed and documented by comparing it with the autofluorescence emitted by chloroplasts.

### Quantification of SL levels

The roots and tiller nodes of Col-0, *Atd27* mutants, and homozygous overexpression lines in different backgrounds were hydroponically cultivated using a Hoagland nutrient solution with a 1/8 dilution ratio for ∼3 weeks until reaching the tillering stage, with each sample being replicated 3 times. After cultivation, tissues were collected from the field, rinsed with deionized water, dried, and subsequently pulverized in liquid nitrogen. Approximately 0.5 g of the test sample was weighed into a test tube, followed by the addition of 5 mL acetonitrile solution for overnight extraction at 4°C. Subsequently, samples were centrifuged at a speed of 12,000 r min^−1^ for 5 min, after which the resulting supernatant was collected and put to the side. A 5-fold volume of acetonitrile solution was added to the precipitate to elucidate 2 further extractions. Subsequently, all the above supernatants were recombined. Following the addition of 200 mg C18 filler, vigorous shaking for 30 s ensued, followed by centrifugation at 10,000 r min^−1^ for 5 min to obtain the supernatant. After concentration, the supernatant was redissolved in 300 µL methanol and filtered through a 0.22 µm organic phase filter membrane. Subsequently, the sample was subjected to detection using ultra-performance liquid chromatography coupled with tandem mass spectrometry [UPLC–MS/MS (QTRAP6500, AB)]. The reagents utilized in the test included 5-Deoxystrigol [5DS (Anpel, CAS: 151716-18-6)], GR24 (Yuan Ye, Shanghai, CAS: 76974-79-3), strigol (Anpel, CAS: 1820-11-2), acetonitrile and methanol: HPLC (Merck Company), and CNW C18 QuEChers filler (Anpel Company, Shanghai). The chromatographic column employed was Agilent Poroshell120 SB-C18 (2.1 × 150 mm, 2.7µm) with a column temperature of 30°C. Mobile phase A consisted of a mixture of water containing 0.1% formic acid, while mobile phase B comprised acetonitrile; the flow was set at 0.3 mL min^−1^ [for detailed test conditions, according to the method described previously (Besserer *et al.* 2006; Jamil *et al.* 2010; Proust *et al.* 2011)].

### Statistical analysis

The comparison between a treatment and a control was conducted using *t*-tests (and nonparametric tests). For comparisons between multiple treatments and a control, one-way 2-tailed ANOVAs were performed as described in the figure legends. All data analysis was conducted using GraphPad Prism 9.5 software.

## Results

### *PjD27* downregulated the tiller number of *P. juncea*

**Fig. 1.**
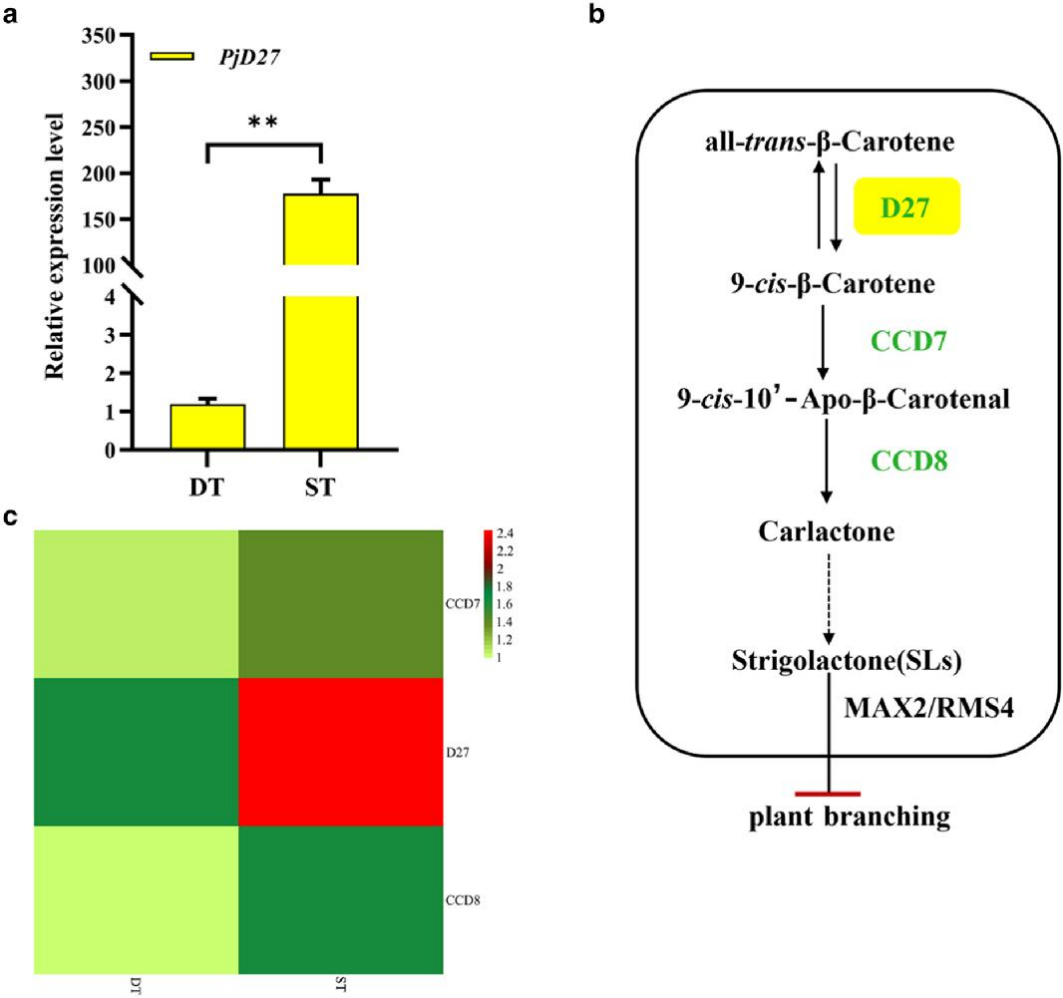
Regulation of *PjD27* on tiller number of *P. juncea*. a) Expression of *PjD27* quantified by qRT-PCR analysis. DT represents dense tiller and ST represents sparse tiller in *P. juncea*. Asterisks denote significant differences between the ST and DT [**P < 0.01, *t*-tests (and nonparametric tests)]. b) D27 is involved in the synthesis of SL and regulates the growth of plant branches. The solid arrow indicates the reaction direction of each step, and the virtual arrow indicates the reaction direction of omitting some steps, and the horizontal bar indicates the inhibition. c) Expression of DEGs related to the biosynthesis of SLs.

In order to verify the expression level of *PjD27* gene in sparse tiller and DT individuals of *P. juncea*, we used qRT-PCR using the cDNA of *P. juncea* at the tillering stage as a template. The results showed that the expression level of *PjD27* in ST was significantly higher than that in DT (Fig. 1a), indicating that *PjD27* downregulated the number of tillers in *P. juncea*. This result is consistent with the findings from transcriptome analysis. Simultaneously, we observed that CCD7 (BMK_Unigene_196444) and CCD8 (BMK_Unigene_152703), which are located downstream of D27 (BMK_Unigene_142326), exhibited a downregulatory effect on the tiller number of *P. juncea* (Fig. 1c). In order to further insight into the effect of *PjD27* on tillering regulation, we performed a pathway analysis of PjD27 based on the research results of KEGG database (Li, Yun *et al.* 2022) and others (Brewer *et al.* 2009; Dun *et al.* 2009). It is speculated that PjD27 was involved in the biosynthesis of SLs, thereby inhibiting plant branching (Fig. 1b).

### Evolutionary relationship analysis of *D27* genes

**Fig. 2.**
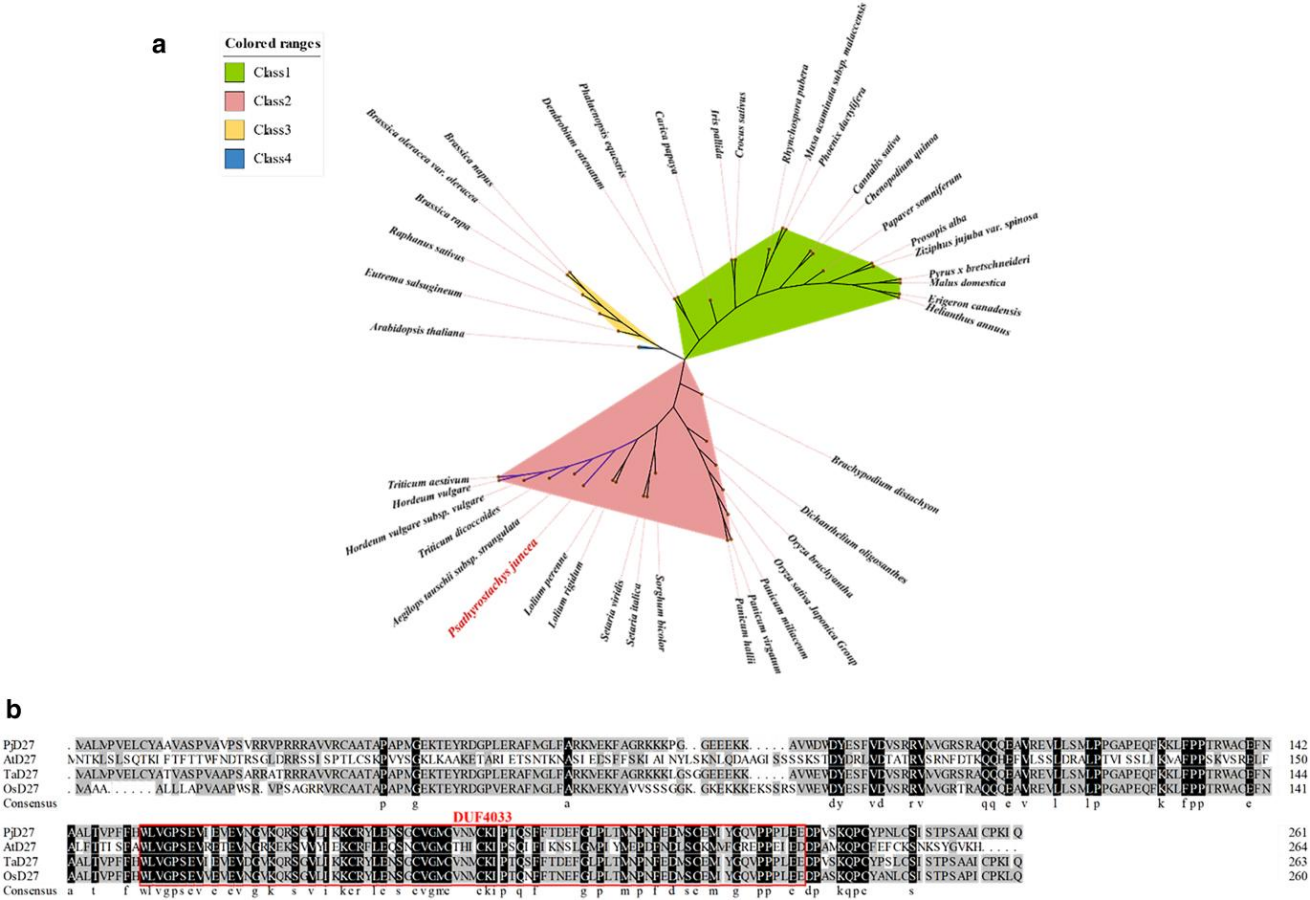
Sequence and phylogenetic analysis of D27. a) Phylogenetic tree of *D27* genes from different species. The phylogenetic tree was built using the NJ method with 1,000 bootstrap replications. Arabic numerals (1–4) represent each gene cluster, which are labeled with different colors. *P. juncea* has been marked in red (a). Purple lines represent a subclass of *P. juncea* and its highly homologous species. b) Multiple alignments of D27proteins in different species. The red rectangle denotes the conserved domain of DUF4033.*Pj*, *P. juncea*, *At*, *A. thaliana*, *Ta*, *Triticum aestivum*, *Os*, *Oryza sativa* (here specifically denotes *Oryza sativa Japonica Group*).

To study the evolutionary relationships among the *D27* genes in different species, 41 *D27* genes were analyzed to construct an unrooted phylogenetic tree. The *D27* genes were classified into 4 groups (Fig. 2a; Supplementary Table 1), which contained 17 (class 1), 18 (class 2), 6 (class 3), and 1 (class 4) members. Our phylogenetic tree suggested that *D27* genes evolved to different degrees in the evolutionary process. The *D27* genes of *P. juncea*, wheat, and other *Triticeae* crops were divided into the same subclass, indicating that they are highly homologous. As further evidence of their shared ancestry, we point out that the PjD27 protein in *P. juncea* had the same highly conserved domain, named DUF4033, as D27 proteins in *Arabidopsis*, wheat and rice, suggesting that they were orthologues of PjD27 protein (Fig. 2b). We present this comparison as theoretical evidence for the functional role *PjD27* likely plays in *P. juncea*.

### *PjD27* is necessary for regulating the number of plant branches

**Fig. 3.**
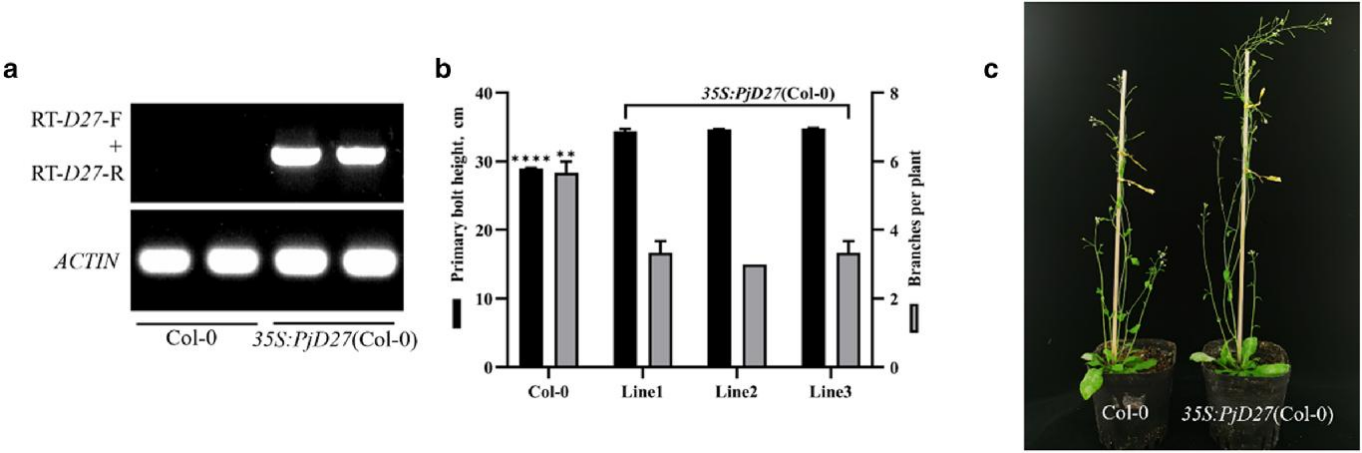
Overexpression of *PjD27* gene downregulates the number of branches in *Arabidopsis*. a) RT-PCR analysis of *PjD27* transcripts in seedlings homozygous. Primers were designed in the CDS of *PjD27* gene. Col-0 serves as a control for RT. b) Branch numbers (dark gray bars) and plant height (black bars) in 42-day-old Col-0 and 3 independent *PjD27*-OE transgenic *Arabidopsis* lines. Data are means ± SE (*n* = 30 plants). Asterisks denote significant differences between treated and untreated samples within the same genotype (**P < 0.01, ****P < 0.0001, one-way ANOVA). c) Axillary branching in the representative plants described in a and b.

Previous studies have shown that the *Arabidopsis d27* mutant plants had a significantly increased branching phenotype (Waters *et al.* 2012). To verify the biological function of the *PjD27*, we first introduced the *35S:PjD27* vector into Col-0 plants. RT-PCR analysis showed that introduction of the *35S:PjD27* vector led to the development of complete *PjD27* transcripts in *Arabidopsis* (Fig. 3a). Through the phenotypic analysis of 3 independent *PjD27*-OE transgenic *Arabidopsis* lines (Supplementary Fig. 2a) and Col-0, it was found that the increased expression of *PjD27* caused an obvious decrease in branches and increase in primary bolt height in the *PjD27*-OE lines compared with the wild type (Fig. 3, b and c). We took this as evidence that *PjD27* plays an important role in regulating the number of plant branches.

**Fig. 4.**
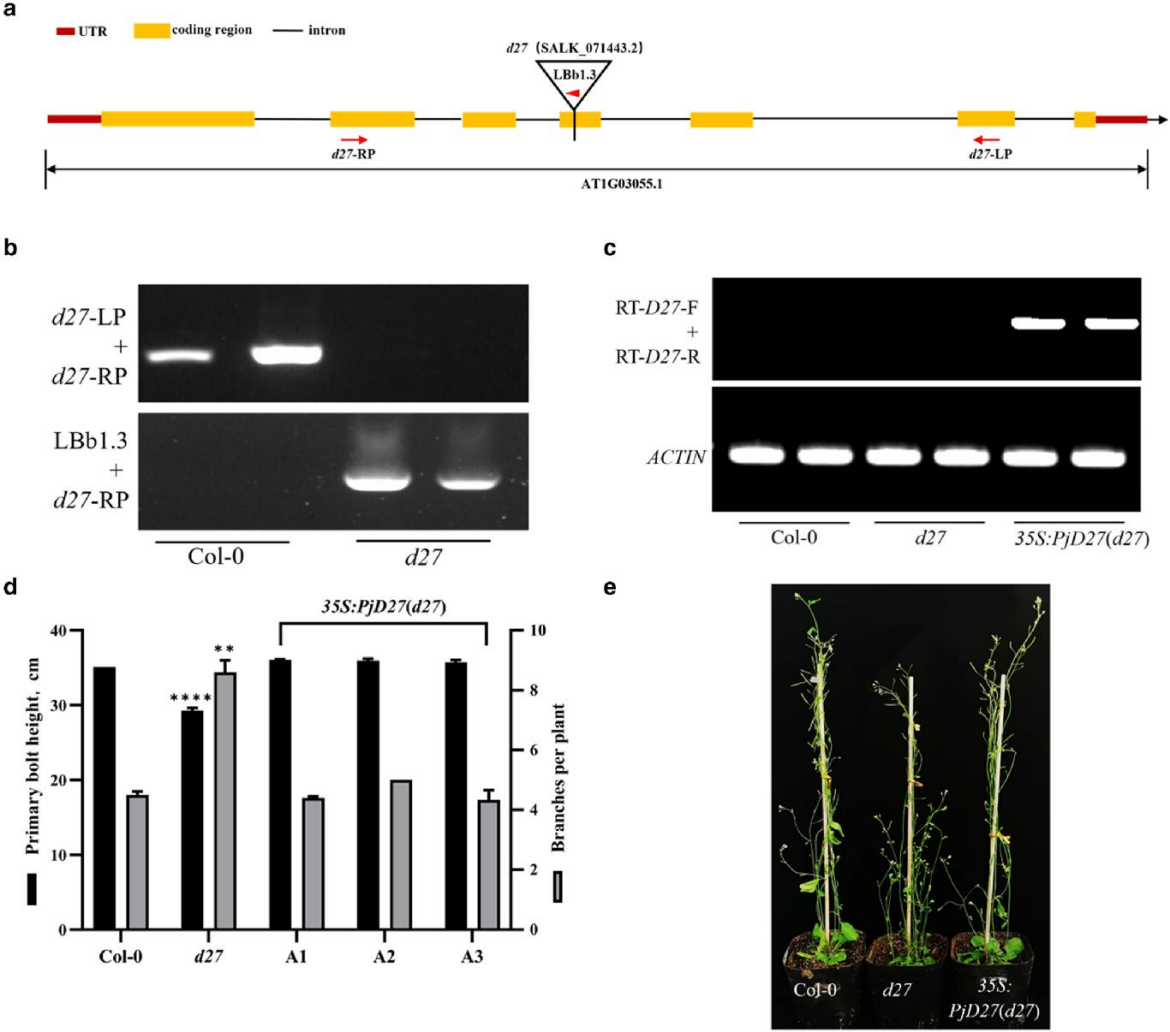
The expression of *PjD27* in *Atd27* plants completely restored the phenotype back to that of the wild type. a) The primary transcript structure of the *Arabidopsis d27*. Inverted triangle indicates the position of T-DNA insertion in *Atd27*, and arrows represent the approximate positions and orientations of PCR primers. UTR, untranslated region. b) RT-PCR analysis of *Atd27* transcripts in seedlings homozygous for T-DNA insertion allele. Primers were selected to span insertion point and an upstream region of the transcript. c) RT-PCR verification of *35S:PjD27*(*d27*) plants. The primer pair was designed based on the coding region sequence of *PjD27*. d) Branch numbers (dark gray bars) and plant height (black bars) in 42-day-old Col-0, *Atd27* mutant plants and 3 independent *PjD27*-OE transgenic *Arabidopsis* complementary lines. Data are means ± SE (*n* = 30 plants). d) Asterisks denote significant differences that *Atd27* mutant plants compared with Col-0 and complementary lines (**P < 0.01, ****P < 0.0001, one-way ANOVA). e) Phenotypes of representative plants described in c and d.

To further understand the function of *PjD27* in plant branches, we also introduced the *35S:PjD27* vector into homozygous *Arabidopsis d27* mutant plants (Fig. 4, a and b), and we complemented the mutant with a cDNA encoding the PjD27 protein, but designed the cDNA so that the PjD27 protein would only be expressed under the control of the cauliflower mosaic virus 35S promoter. Three independent transgenic complementary lines (*35S:PjD27*-A1, *35S:PjD27*-A2, and *35S:PjD27*-A3) were selected for phenotypic analysis (Supplementary Fig. 2b). The result showed that there were no phenotypic differences in plant branches and plant height between the 3 independent transgenic complementary lines and Col-0, and an increased branching and severe dwarf phenotype in *Arabidopsis d27* mutant plants was complemented by *35S:PjD27* (Fig. 4, d and e). At the same time, RT-PCR analysis also showed *Arabidopsis d27* mutant plants had accumulated *PjD27* transcripts (Fig. 4c). The biological role of the *PjD27* gene in the regulation of branch number and plant height in *Arabidopsis* was consistent with the role that the *OsD27* gene plays in rice (Lin *et al.* 2009). We took this experimental result as further evidence that *PjD27* likely plays a similar role in regulating the branch number and forage yield of *P. juncea.*

### Western blotting and subcellular location of PjD27 protein

**Fig. 5.**
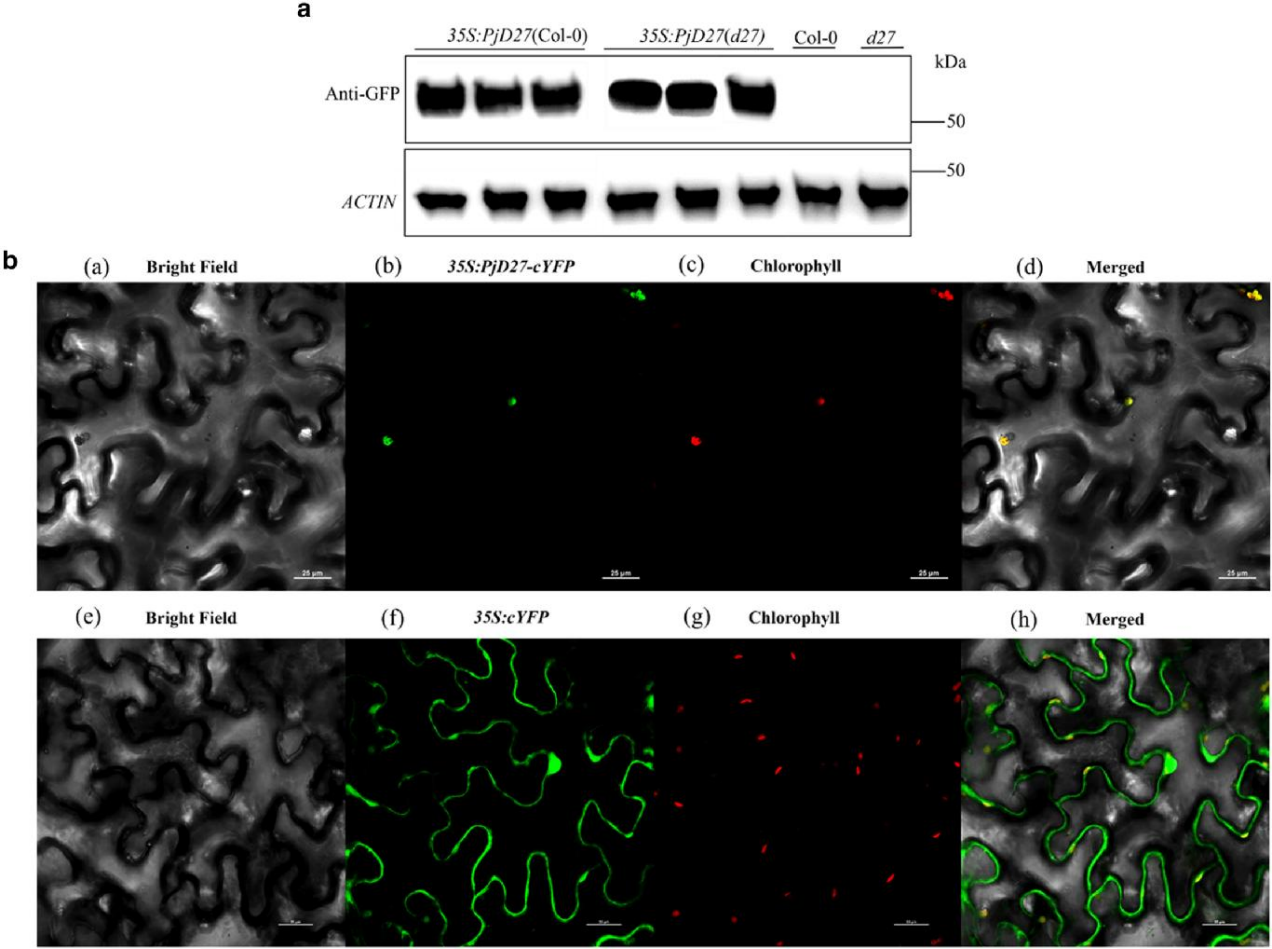
Overexpression characteristics of PjD27 protein. a) Western blotting verification of PjD27. The six protein bands are 6 independent transgenic lines. b) Subcellular localization analysis of PjD27-cYFP fusion protein. b, PjD27-cYFP fusion protein fluorescence, f, 35S: p1300-cYFP fluorescence; c and g, chloroplast autofluorescence; d and h, the merged images denote colocalization of the 2 fluorescent signals; a and e, the cell outline of tobacco in bright field micrographs of the same field of view. The scale size in the picture is 25µm.

We observed that *PjD27* was expressed in all the *35S:PjD27* lines, while the PjD27 protein was not expressed in Col-0 and *Atd27* mutant plants. To further explore the subcellular location of the PjD27, we generated a chimeric construct encoding the full-length PjD27 protein fused with YFP. Following transient expression in tobacco, imaging of fluorescence showed that the YFP fluorescence fully overlapped with the red fluorescence associated with chloroplasts (Fig. 5b). The result showed that the PjD27-cYFP fusion protein was predominantly localized in the chloroplast, which was consistent with the cellular location of OsD27 in rice (Lin *et al.* 2009) and TaD27-B in wheat (Zhao *et al.* 2020).

### The expression level of *PjD27* in *Arabidopsis* is significantly increased

**Fig. 6.**
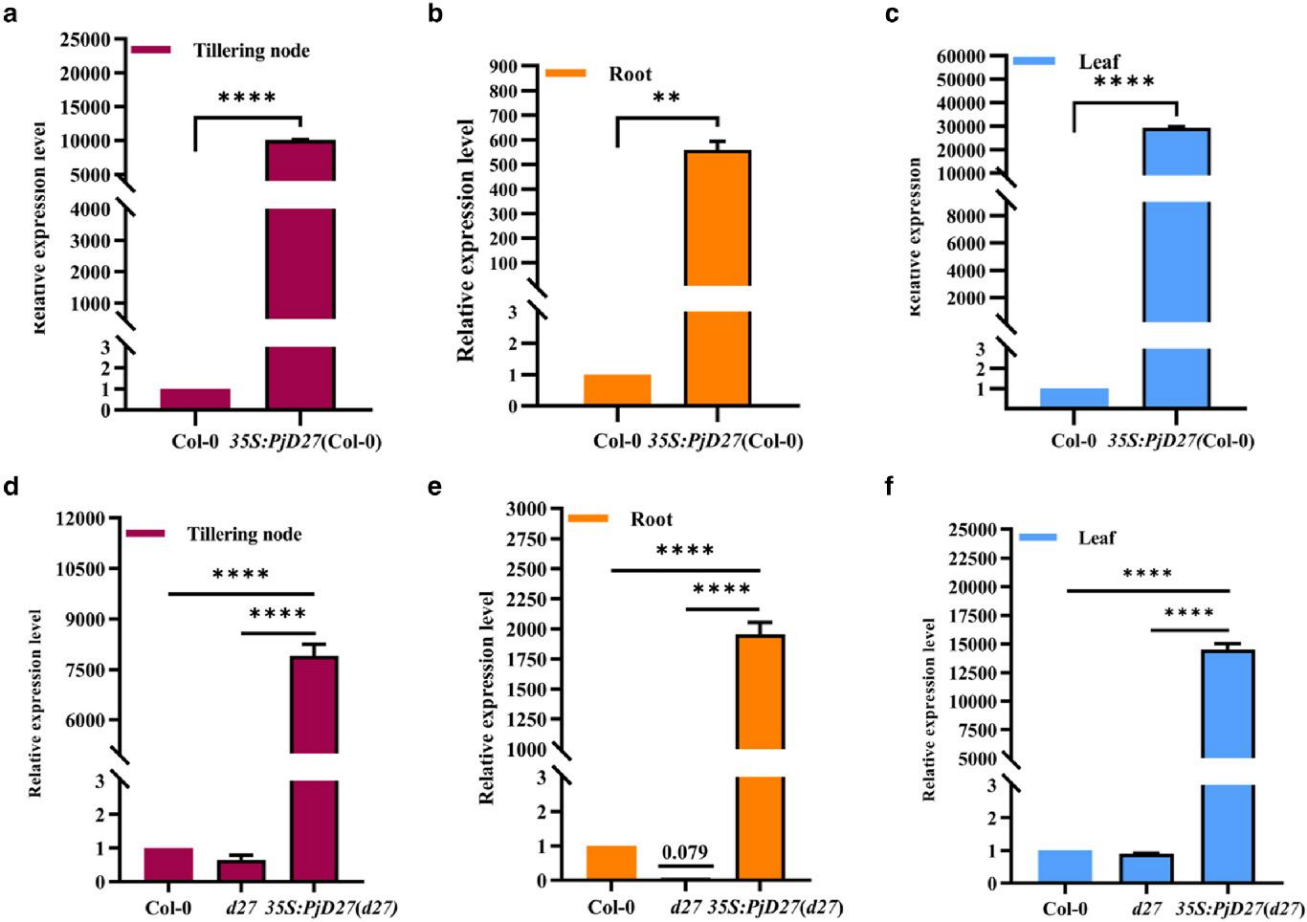
Expression level of *PjD27* in different tissues of *Arabidopsis*. a–c) The analysis of relative expression level of *PjD27* in different tissues of 3*5S:PjD27*(Col-0) plants compared with Col-0. d–f) The analysis of relative expression level of *PjD27* in different tissues of 3*5S:PjD27*(*d27*) plants compared with Col-0 and *Arabidopsis d27* mutant plants. Transcripts were normalized to *Actin* gene (*At5g09810)*. The expression level of *PjD27* in Col-0 was normalized to 1, which was scaled to the value in the remaining samples. All the templates were the cDNAs of roots, tiller nodes, and leaves of 32-day-old *Arabidopsis*. The data represent the results of quantitative analysis obtained from 3 biological replicates and 3 technical replicates for each sample. Asterisks denote significant differences between Col-0 and *PjD27*-OE transgenic *Arabidopsis* lines, as well as between complementary lines compared to Col-0 and *Atd27* mutant plants (**P < 0.01, ****P < 0.0001, one-way ANOVA).

The primers for qRT-PCR were designed based on the coding region sequence of *PjD27*. qRT-PCR was performed using cDNAs from roots, tillering nodes, and leaves of Col-0, *Arabidopsis d27* mutant plants, and *PjD27*-OE transgenic *Arabidopsis* lines to analyze the expression level of the *PjD27* gene in each set of plants. We observed that the *PjD27* transcript level was significantly increased in tillering nodes (Fig. 6a), roots (Fig. 6b), and leaves (Fig. 6c) of 32-day-old *PjD27*-OE transgenic *Arabidopsis* lines compared with the wild type. The expression level of the *PjD27* gene in leaves and tillering nodes was orders of magnitude (10^3^ to 10^4^) times greater than expressed in roots. When the cDNA encoding the PjD27 protein complemented *Arabidopsis d27* mutant plants, the expression level of the *PjD27* gene was also markedly increased relative to the Col-0 and *Arabidopsis d27* mutant plants (Fig. 6, d–f).

### D27 protein feedback to SL

**Fig. 7.**
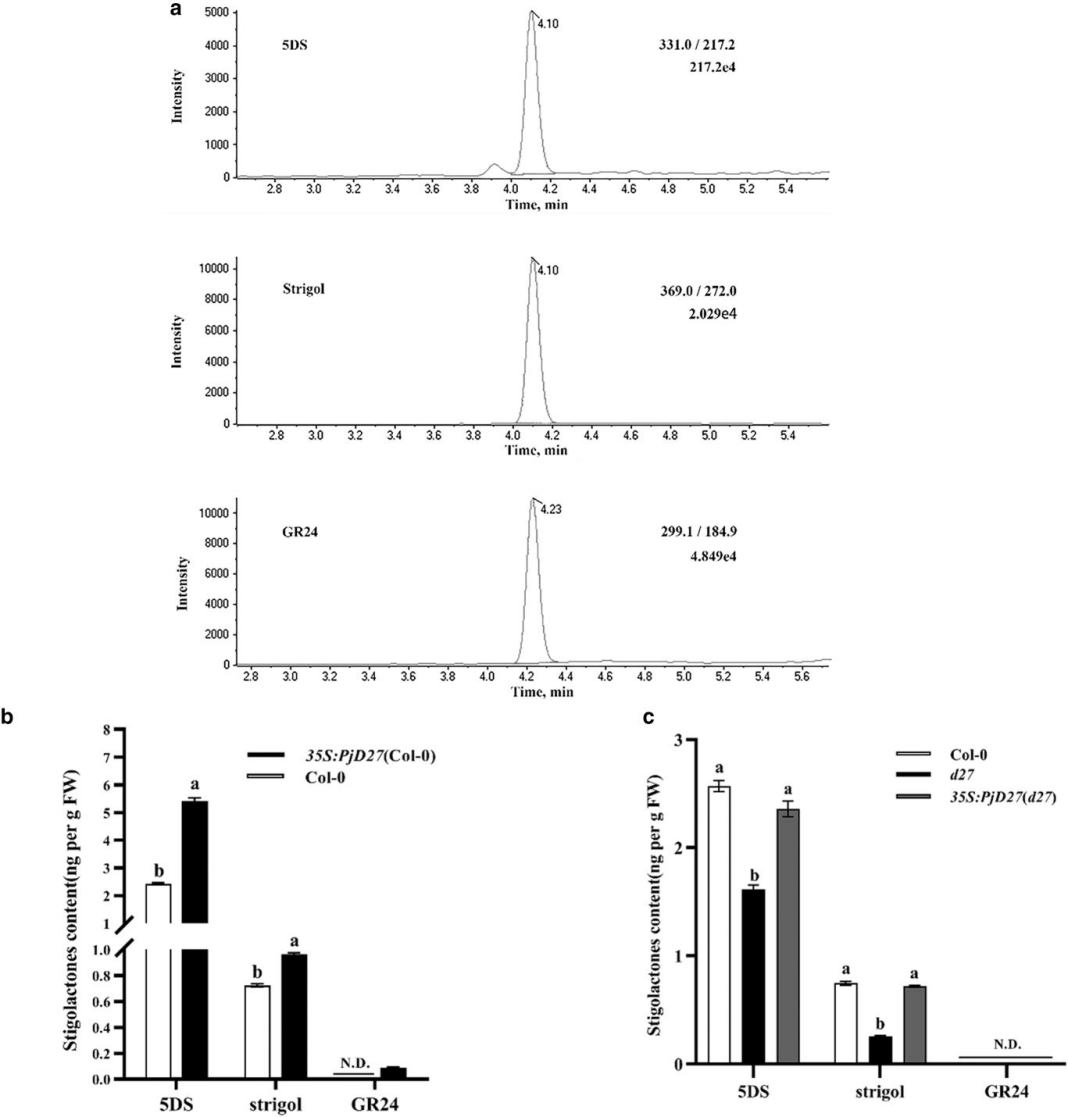
SL analysis in *Arabidopsis.* a) UPLC–MS/MS chromatograms for internal standard of 5DS, Strigol and GR24. b) UPLC–MS/MS analysis of SLs levels in wild-type (Col-0) and *35S:PjD27*(Col-0) plants (mean + s.d., *n* = 3). FW, fresh weight. c) UPLC–MS/MS analysis of SLs levels in wild-type (Col-0), *Atd27* mutants, and *35S:PjD27*(*d27*) plants (mean + s.d., *n* = 3). FW, fresh weight. Different letters indicate significant differences (*P* < 0.05, one-way ANOVA). N.D. denotes that the SL content was either not detected or below the detection limit.

To explore the potential role of *PjD27* in SL biosynthesis, we analyzed SLs in tillering nodes and root tissues of *35S:PjD27* plants by UPLC–MS/MS. We used 3 common standards—2 SLs (5DS and strigol), and the synthetic compound GR24 (frequently used as an analog to conventional SLs), as internal standards for quantification to investigate the correlation between *PjD27* expression and different types of SLs in *Arabidopsis*. We selected respectively m/z 331, 369, and 299.1 for 5DS, strigol, and GR24, as parent ions on quadruple mass spectrometry and detected respectively m/z 217.2, 272, and 184.9 for 5DS, strigol and GR24, as fragment ions on time-of-flight mass spectrometry after collision-induced dissociation for quantification (Fig. 7a). To determine whether SLs participate in the branching inhibitor pathway in *Arabidopsis*, we examined the effect of 5DS, strigol and GR24 on the branching phenotype of Col-0, *35S:PjD27* (Col-0), *Atd27* mutants and *35S:PjD27* (*d27*).

We observed that the levels of 5DS in *35S:PjD27* (Col-0) plants were significantly elevated compared with the wild type. Furthermore, quantitative analysis of strigol and GR24 levels also revealed a higher production of SLs in *35S:PjD27* (Col-0) plants compared to Col-0 (Fig. 7b). Upon complementation of *AtD27* mutants with a cDNA encoding the PjD27 protein, we analyzed the levels of SLs in *35S:PjD27* (*d27*) plants, *Atd27* mutants, and Col-0. Remarkably, SL levels in *35S:PjD27* (*d27*) plants were restored to those observed in Col-0, both exhibiting significantly higher levels compared to *Atd27* mutants (Fig. 7c). Among the SLs observed in plants, 5DS content was highest, suggesting a predominant involvement of PjD27 in regulating plant branching through 5DS biosynthesis in *Arabidopsis*. However, the presence of GR24 was either undetected or below the detection limit. These results indicate that the overexpression of the *PjD27* gene leads to an increase in overall SL levels in *Arabidopsis*.

## Discussion

Previous research has employed forward genetics to identify tiller-related genes in different phenotypes of *P. juncea* (Li, Yun *et al.* 2022). This study uses a revere genetics approach to the candidate gene *PjD27* to infer the role it plays in tillering in *P. juncea*. Prior to that, to further elucidate the regulatory role of *PjD27* in the shoot branching of *P. juncea*, we conducted an analysis to determine the expression level of *PjD27* in both dense and sparse tiller samples during the vigorous tillering stage. The finding was consistent with the result obtained from transcriptome analysis using the FPKM method, indicating that *PjD27* downregulated the tiller number of *P. juncea* (Fig. 1, a and c). Through a conserved domain analysis, we identified a highly conserved domain-containing protein, known as DUF4033, within the PjD27 protein. This domain-containing protein is also present in D27 proteins found in wheat, rice, and *Arabidopsis*, suggesting that they were orthologues of the PjD27 protein (Fig. 2b).

We felt this was sufficient evidence that *PjD27* would be related to tillering in *P. juncea*. Phenotypic analysis showed the number of branches and plant height of *PjD27*-OE transgenic *Arabidopsis* lines were markedly changed (Fig. 3). In addition, the expression of *PjD27* in *Arabidopsis d27* mutant plants almost completely restored the phenotype back to that of the Col-0 (Fig. 4). The *35S:PjD27* (Col-0) plants exhibited a reduced branching phenotype similar to that of *TaD27-B*-OE plants in wheat, when compared with wild-type plants. The complementation function of *35S:PjD27* (*d27*) was consistent with that of *TaD27-B* in *Atd27* plants. We found this to be evidence that *PjD27* is a functional ortholog of *d27* in *Arabidopsis* (Zhao *et al.* 2020). *D27* is well established as playing a pivotal role in regulating the number of plant branches and possesses a conserved function in suppressing branch formation in both monocotyledonous and dicotyledonous plants (Lin *et al.* 2009).

The expression of the PjD27 protein in *Arabidopsis* materials, including the wild-type and *Atd27* mutants, was analyzed using a WB (Fig. 5a). The PjD27 protein was robustly expressed in *Arabidopsis* and was associated with significant changes in plant height and branch number in the plant's phenotype (Figs. 3 and 4).

The subcellular location of proteins is intricately linked to their biological functions (Martin-Merchan *et al.* 2023). The OsPAP3 protein of rice is located within the chloroplast nucleoids, exhibiting a close association with the abundance of chloroplasts. The overexpression of the *OsPAP3* gene in plants led to an increase in both chloroplast abundance and tiller number, thereby playing a pivotal role in enhancing rice yield (Seo *et al.* 2024). To investigate the function of the PjD27 protein, we conducted a study on its subcellular location in tobacco through transient transfection (Fig. 5b). We observed that—similar to *OsPAP3* in rice—the subcellular location of the PjD27 protein is exclusively confined to the chloroplast, a pivotal organelle crucial for optimal plant growth (Fan *et al.* 2023). Deciphering the precise subcellular location of the PjD27 protein provides valuable insights into unraveling the underlying mechanism governing tillering in *P. juncea*.

RT-qPCR can rapidly and accurately validate gene expression results at the transcriptional level (Liu *et al.* 2022; Yang *et al.* 2022). The analysis of gene expression can reflect the impact of genes on the physiological state of plants (Asif *et al.* 2023). The expression level of *PjD27* gene exhibited variation across different plant tissues in *PjD27*-OE transgenic *Arabidopsis* plants. Specifically, the *PjD27* transcript levels in tillering nodes and leaves were significantly higher compared to those in roots, indicating a correlation between tissue-specific expression and gene function (Qi *et al.* 2023). The *PjD27* gene was implicated in the regulation of branch numbers in *Arabidopsis*. Its specific expression in tillering nodes may be associated with its role in regulating tillering, while the increased expression in leaves could be attributed to the location of the PjD27 protein in chloroplasts.

Numerous studies have consistently demonstrated that D27 primarily participates in the biosynthesis of SLs and exerts a pivotal role in the regulation of plant branching (Lin *et al.* 2009; Xie *et al.* 2010; Harrison *et al.* 2015; Wang, Tu *et al.* 2023; Wang, Yang *et al.* 2023). Our findings demonstrate the involvement of PjD27 in SL biosynthesis (Fig. 7), thereby regulating branching in *Arabidopsis*, analogous to the regulatory roles of pea (*P. sativum* L.) CCD8 (Gomez-Roldan *et al.* 2008) and rice d17 (Chen *et al.* 2023). The *CCD8* mutant in peas almost completely restored the phenotype back to that of the wild type through the exogenously applied SL-related inhibitor. Furthermore, we observed that our samples had high levels of 5DS, consistent with phosphorus-deficient *d3*-1, *d10*-1, and *d17*-1 rice mutants, suggesting 5DS plays an inhibitory role in *Arabidopsis* and *P. juncea* (Umehara *et al.* 2008). Several studies have demonstrated the inhibitory effects of naturally occurring SLs, namely strigol and 5DS, as well as the SL analog GR24 and its downstream metabolites, on branching processes (Burger 2022; Yamaguchi *et al.* 2023), which further substantiates our research findings. The determined content of SLs in our research results was relatively low, potentially due to the need for further optimization of sampling time and extraction methods. Nevertheless, the discovery regarding PjD27's involvement in regulating plant branching through its participation in SL biosynthesis establishes a strong theoretical basis and provides valuable experimental resources for subsequent studies on tillering regulation in *P. juncea*.

Distant hybridization is an important approach for broadening the genetic base of plant cultivars. In many breeding programs, researchers will transfer genes that improve plant productivity from wild relatives to the plant (Kong *et al.* 2018). For example, a wheat-*Haynaldia villosa* derived line carried 2 translocations: 6VS/6AL and 4VS/4DL resisted powdery mildew and stripe mosaic virus, respectively (Chen *et al.* 1995; Zhang *et al.* 2005). Barley (*Hordeum vulgare*) yellow dwarf viruses (BYDVs) result in considerable yield losses in cereals. In order to solve this problem, the genome-wide miRNA expression profiling in *P. huashanica was* analyzed, which revealed the resistance response of miRNAs to *BYDV*-GAV infection (Shen and Li 2022). *P. juncea* is a perennial Triticeae forage that can restrain weeds. *Halogeton glomeratus* is a particularly difficult weed to manage. However, the planting of *P. juncea* and forage kochia (*Bassia prostrata*) can reduce halogeton frequency, thereby providing an opportunity for the survival of cultivated plants (Smith *et al.* 2016). In addition, *P. juncea* also has an excellent gene pool and is a potential resource for the genetic improvement of wheat (Dong *et al.* 2008). For example, the novel D-hordeins encoded by *Ns 1.3*, *Ns 2.6*, and *Ns 2.9* genes in *P. juncea* have a very important potential for enhancing the end-use quality of wheat flour. Furthermore, these D-hordeins can also contribute to the understanding of *Triticeae* prolamins’ evolution (Hu *et al.* 2018). Therefore, the utilization prospects of *P. juncea* for wheat improvement are promising.

In summary, *PjD27* represents a new genetic tool for exploring shoot branching mechanisms in *P. juncea*, and it plays an important role in cultivating high-yield *P. juncea*. Furthermore, functional analysis of the *PjD27* gene also contributes to promoting high-quality wheat production.

## Conclusion

The present study demonstrates that PjD27 plays critical roles in regulating *Arabidopsis* branching number by participating in SL biosynthesis. Thus, PjD27 is a functional orthologue of *D27* in *Arabidopsis*. In addition, we also found that the PjD27 protein was mainly localized in the chloroplast, suggesting a potential functional association with tillering similar to that observed in other chloroplast-localized proteins. The functional analysis of the *PjD27* gene can provide a theoretical basis for multitillering germplasm creation in *P. juncea* and hold significant research implications for enhancing grain yield in *P. juncea*.

## Supplementary Material

jkae147_Supplementary_Data

## Data Availability

Strains and plasmids are available upon request. The Sequence data are available at GenBank; the accession numbers are listed in Supplementary Table 1. The addresses are as follows: https://www.ncbi.nlm.nih.gov/search/all/?term=OR865585, and the amino acid sequence is translated from the online website ORFfinder Home—NCBI (nih.gov). The raw sequencing reads of transcriptome data in this study are available in Sequence Read Archive database (accession number PRJNA789128). The addresses are as follows: (https://www.ncbi.nlm.nih.gov/bioproject/PRJNA789128/). Supplemental material available at G3 online.
