# Supplementary material for: The Psathyrostachys juncea DWARF27 gene encodes an all-trans-/9-cis-beta-carotene isomerase in the control of plant branches in Arabidopsis thaliana by strigolactones
Source: G3 (Bethesda). 2024 Jul 15;14(9):jkae147. doi: 10.1093/g3journal/jkae147 (PMC11373637; doi:10.1093/g3journal/jkae147)
Supplement: jkae147_Supplementary_Data [file jkae147_supplementary_data.docx]

Supplementary Material

| **Table S1. Amino acid sequence information of D27 proteins in different species** | | | |
| --- | --- | --- | --- |
| **Species names** | **Sequence version** | **Species names** | **Sequence version** |
| ***Arabidopsis thaliana*** | ***At1g03055*** | ***Brassica oleracea var. oleracea*** | **XP_013608610.1** |
| ***Triticum dicoccoides*** | **XP_037460112.1** | ***Brassica rapa*** | **XP_009112920.1** |
| ***Aegilops tauschii* subsp*. strangulata*** | **XP_020156927.1** | ***Hordeum vulgare* subsp*. vulgare*** | **XP_044957629.1** |
| ***Triticum aestivum*** | **XP_044433577.1** | ***Brachypodium distachyon*** | **XP_003573300.1** |
| ***Lolium perenne*** | **XP_051200690.1** | ***Dendrobium catenatum*** | **XP_020691525.1** |
| ***Lolium rigidum*** | **XP_047080210.1** | ***Raphanus sativus*** | **XP_018455679.1** |
| ***Hordeum vulgare*** | **KAE8770425.1** | ***Ziziphus jujuba var. spinosa*** | **XP_015896339.2** |
| ***Panicum miliaceum*** | **RLM62170.1** | ***Malus domestica*** | **XP_028948936.1** |
| ***Panicum hallii*** | **XP_025820350.1** | ***Rhynchospora pubera*** | **KAJ4803776.1** |
| ***Setaria viridis*** | **XP_034598954.1** | ***Helianthus annuus*** | **XP_022029371.1** |
| ***Panicum virgatum*** | **XP_039852580.1** | ***Prosopis alba*** | **XP_028756021.1** |
| ***Setaria italica*** | **XP_012702109.1** | ***Pyrus x bretschneideri*** | **XP_048422660.1** |
| ***Sorghum bicolor*** | **XP_002444917.2** | ***Iris pallida*** | **KAJ6840563.1** |
| ***Dichanthelium oligosanthes*** | **OEL20132.1** | ***Eutrema salsugineum*** | **XP_006391710.1** |
| ***Oryza brachyantha*** | **XP_006659061.2** | ***Erigeron canadensis*** | **XP_043628432.1** |
| ***Oryza sativa Japonica Group*** | **XP_015648697.1** | ***Carica papaya*** | **XP_021905140.1** |
| ***Crocus sativus*** | **UXN85564.1** | ***Chenopodium quinoa*** | **XP_021757762.1** |
| ***Phalaenopsis equestris*** | **XP_020589137.1** | ***Musa acuminata* subsp*. malaccensis*** | **XP_009391936.1** |
| ***Phoenix dactylifera*** | **XP_008784872.1** | ***Cannabis sativa*** | **XP_030500161.1** |
| ***Brassica napus*** | **XP_013686132.1** | ***Papaver somniferum*** | **XP_026441249.1** |
| ***Psathyrostachys juncea*** | **OR865585** |  |  |

**Note:**The Sequence version of *Psathyrostachys juncea* represents the nucleotide sequence login number information (The addresses are as follows: <https://www.ncbi.nlm.nih.gov/search/all/?term=OR865585> , and the amino acid sequence is translated from the online website [ORFfinder Home - NCBI (nih.gov)](https://www.ncbi.nlm.nih.gov/orffinder/).

| **Table S2. Primers used for qRT-PCR** | | | |
| --- | --- | --- | --- |
| **Name** | **AGI number** | **Forward primer(5'~3')** | **Reverse primer(5'~3')** |
| ***ACTIN-Pj*** |  | **TGGTATGGAAGCTGCTGGAA** | **TCAGCAATACCCGGGAACAT** |
| ***ACTIN*** | ***At5g09810*** | **AGTGGTCGTACAACCGGTATTGT** | **GAGGAAGAGCATACCCCTCGTA** |
| **qRT-PCR-*D27*** |  | **AGACAGAGTACAGGGACGGG** | **CTTCCTTCCGGCGAACTTCT** |

**
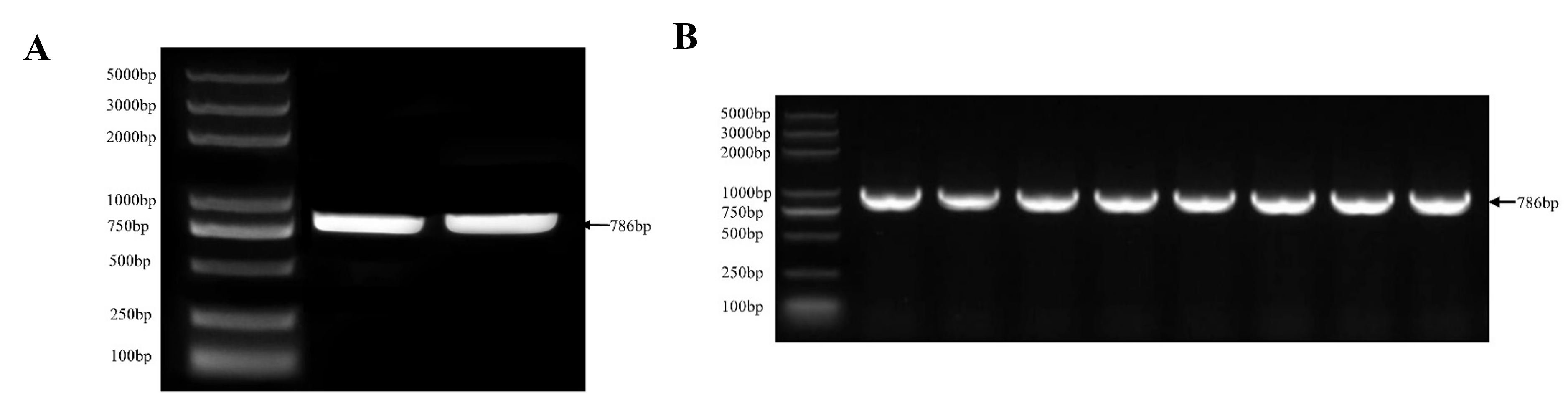
**

**Figure S1.** PCR product of *35S:PjD27* vector construction. PCR product of *PjD27* clone (A). PCR products of positive colonies of *PjD27* (B).


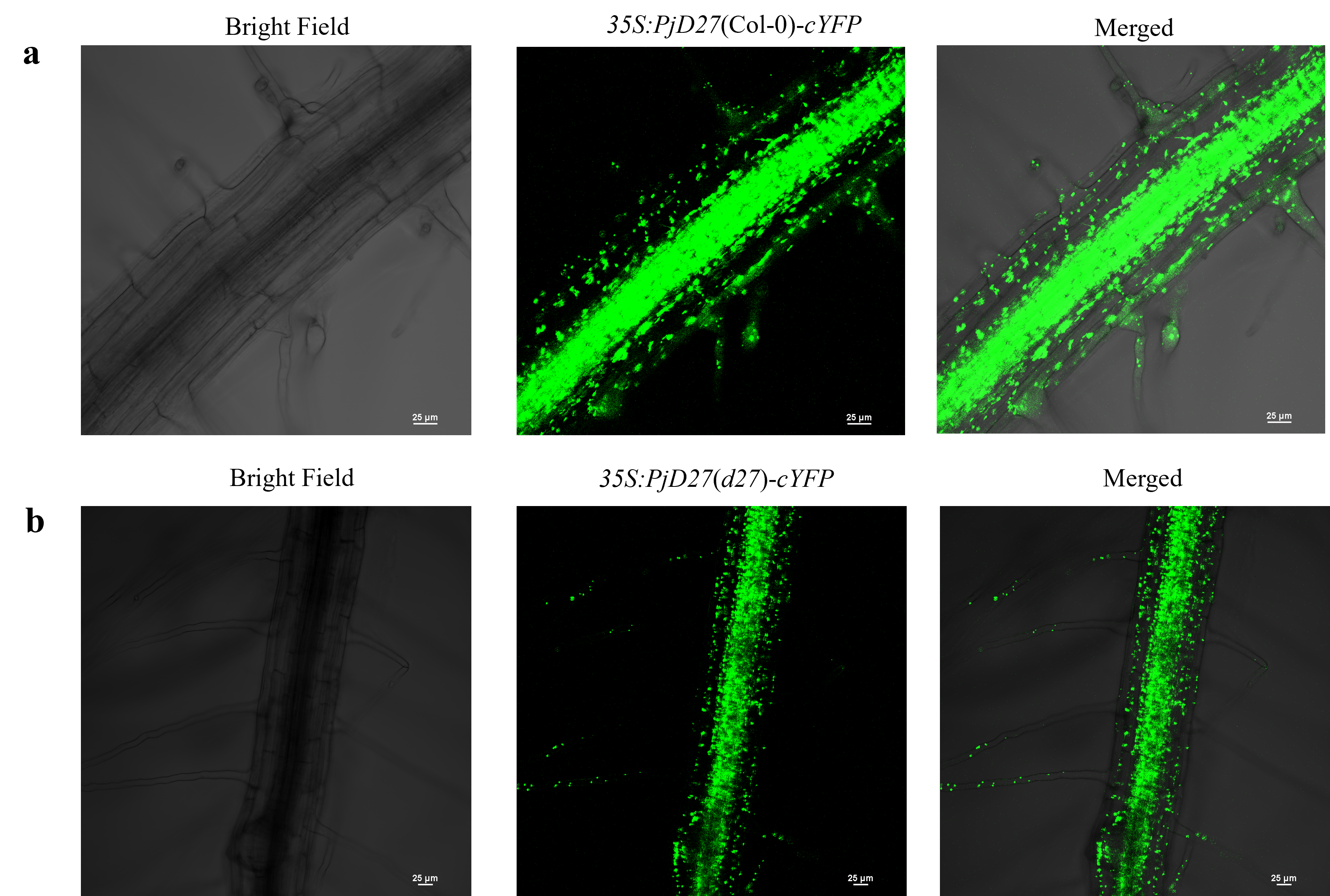


**Figure S2.** Root fluorescence of *35S:PjD27*(Col-0) transgenic lines (a). Root fluorescence of *35S:PjD27*(*d27*) transgenic complementary lines (b).


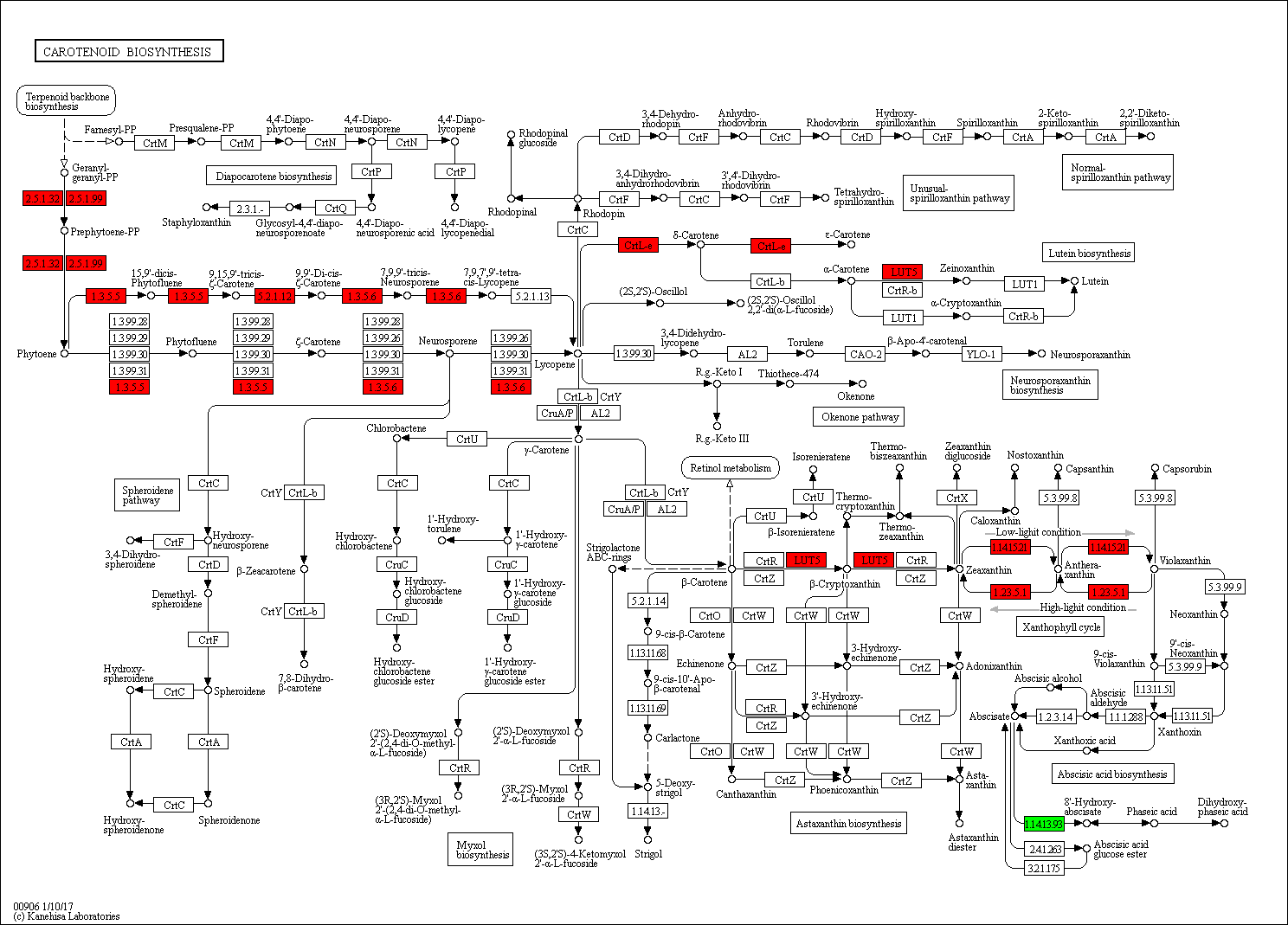


**Figure S3.** Pathway diagram related to *PjD27* gene.
